# Supplementary material for: Deletion of wfs1 Impairs Oligodendrocyte Precursor Cells Dorsal Distribution and Myelination Through the wfs1-hmgcs1 Axis in Zebrafish
Source: Biology (Basel). 2026 Aug 16;15(16):1402. doi: 10.3390/biology15161402 (PMC13510151; doi:10.3390/biology15161402)
Supplement: Supplementary file 1 [file biology-15-01402-s001.zip › File S1-Transcriptome sequencing data and differentially expressed genes (DEGs) in wfs1-- zebrafish at 5 dpf/2.KEGG/wfs1KOvsWT/ALL/src/dre04115.html]

dre04115


- dre:431763

- Up regulated genes

431763(1.93865999132)

- Down regulated genes

431763\_1(-4.20440659354098)

- dre:436699

- Up regulated genes

436699\_1(6.97240743326095)

- Down regulated genes

436699(-8.14472572492325)

- dre:171473

- Up regulated genes

171473(3.06093664837031)

- dre:100330864

- Up regulated genes

100330864(6.66489299727258)

- dre:100320152

- Down regulated genes

100320152(-1.45074630443396)

- dre:796473

- Down regulated genes

796473\_1(-1.57341917911858)

- dre:768248

- Up regulated genes

768248(6.07490717007993)

- dre:561021

- Up regulated genes

561021(2.14284154145004)

- Down regulated genes

561021\_1(-4.68369619674599)

- dre:431763

- Up regulated genes

431763(1.93865999132)

- Down regulated genes

431763\_1(-4.20440659354098)

- dre:140621

- Down regulated genes

140621(-2.68196234303566) 140621\_1(-3.95107590777093)

Close
